# Chatbot Memory: Uncovering How Mental Effort and Chabot Interactions Affect Short-Term Learning

**DOI:** 10.1177/10711813251358242

**Published:** 2025-07-13

**Authors:** Alexandre Marois, Isabelle Lavallée, Gabrielle Boily, Jonay Ramon Alaman, Bérénice Desrosiers, Noémie Lavoie

**Affiliations:** 1 École de Psychologie, Université Laval, Québec, Canada; 2 School of Psychology and Humanities, University of Central Lancashire, Preston, UK; 3 Département de Psychologie, Université de Sherbrooke, Sherbrooke, Canada

**Keywords:** learning, chatbot, artificial intelligence, mental effort, web-based learning

## Abstract

Developments in artificial intelligence (AI) are transforming everyday tasks, including accessing information, learning, and decision making. Generative AI is representative of these changes as it can generate content traditionally reserved for humans with increased efficiency and reduced effort. This includes technologies like ChatGPT and other tools that exploit large language models, typically taking the form of conversational agents (chatbots). These technologies can be useful for self-regulated learning as is the case for Web browsing. It is, however, unclear whether learning with chatbots may be efficient as opposed to other Web-based approaches given the reduced effort related to chatbot interactions. This study assessed how interacting with a chatbot may affect short-term learning and the role of mental effort. Memory performance was equivalent across participants who either interacted with a chatbot or browsed the Internet to find information for answering essay questions. Differences in self-reported workload were, however, found across conditions.

## Introduction

Recent developments in artificial intelligence (AI) and its increasing accessibility are changing our ways of living and working (Dubey et al., 2020). Generative AI is particularly representative of these changes. Such a technology can generate content that is traditionally reserved to humans with increased efficiency and reduced effort. This includes technologies like ChatGPT and other tools that exploit large language models (LLMs), which typically take the form of conversational agents (chatbots) that process natural language and produce responses accordingly (Cao et al., 2023). LLM-based agents have been studied as tools to support knowledge-centered activities such as writing and learning (Rice et al., 2024). Most of this work is focused on assessing how chatbots could promote learning in the context of a classroom or in other structured learning activities (Kohnke et al., 2023). Yet, opportunities for learning with chatbots go beyond scholar uses, including for Web browsing and punctual information research, which involve processes related to self-regulated learning (Al Shloul et al., 2024).

Self-regulated learning has been shown to be involved in computer-based learning environments (Greene et al., 2011). It typically comprises three phases, each relying on different metacognitive strategies that allow managing resources and deploying mental efforts toward specific aspects critical for learning (Zimmerman, 2000). These strategies comprise: (a) a forethought phase, which involves goal setting and strategic planning; (b) a performance phase, which entails metacognitive monitoring and control; and (c) a self-reflection phase, which activates self-evaluation and reflection processes. Across these steps, metacognitive abilities are engaged to manage cognitive effort to dynamically adjust the focus of attention towards and resources assigned to different pieces of information, which in turn affects how said information may be learned. The current article focuses on understanding how using chatbots might impact short-term self-regulated learning and on investigating the interplay with mental effort.

### Chatbot-Supported Learning

A meta-analysis conducted by Wu and Yu (2024) outlined how using chatbots to support education could provide benefits for long-term learning performance, especially among university students. Their results emphasized that chatbots could increase motivation and provide learners with various opportunities. For instance, learners can receive individualized tutoring and elaborate more on their learning by discussing with the AI and receiving personalized feedback. Yet, excessive reliance may also reduce cognitive engagement and long-term retention (Bai et al., 2023). These conclusions are primarily based on studies involving long-term usage (e.g., over multiple weeks during a course). Yet, to the best of our knowledge, very few studies have focused on short-term uses of chatbots for learning.

To better understand what learning mechanisms may be involved during human-chatbot short-term interactions, it is essential to look how mental effort is deployed (Jose et al., 2025). Research shows that encountering certain difficulties that demand mental effort during the learning process can be beneficial (Bjork & Bjork, 2020). These are known as “desirable difficulties.” Desirable difficulties are useful because they activate various encoding and retrieval processes that promote learning, comprehension, and memorization. Typically, learners who encounter too many difficulties in regulating their learning may achieve suboptimal memorization (Bannert et al., 2015; Kizilcec et al., 2017). According to Wang et al. (2019), chatbots can help reduce some undesirable difficulties, namely extraneous cognitive load—that is, task-irrelevant cognitive load—by providing detailed and personalized feedback to learners. Nonetheless, a reduction in mental effort while interacting with a chatbot may also impact other key learning components and even some desirable difficulties given the lack of necessity to actively verify information once the chatbot has provided answers on a given subject. Thus, the cognitive load derived from managing multiple sources of information may actually support learning by promoting self-regulation strategies.

A pilot study conducted by Ju (2023) examined the impact of AI on short-term learning effectiveness. Thirty-two participants were asked to read an article, write a summary, and answer five questions about its content. Three groups were randomly formed: (a) the first group wrote a summary without any help from an AI (manual group); (b) the second group used AI to write the summary (AI group); and (c) the third group engaged in active reading guided by an agent, with the opportunity to ask questions to improve their understanding of the text (active group). The results showed that the exclusive use of AI led to a 25.1% decrease in correct recall compared to the manual group, while partial use of AI (active group) reduced recall by 12%. In addition, the active and AI groups took less time to complete the task than the manual group. It was also observed that the group using AI produced higher-quality summaries within the allotted time. These results suggest that the relationship between humans and AI in the learning process is more effective when it is complementary rather than exclusive, with benefits for answering the essay questions but drawbacks on memory (see also Kosmyna et al., 2025, for evidence of poorer memory recall following the interaction with a chatbot). The short-term effects of chatbots on learning and their interplay with mental effort, however, are still unclear.

### Study Objectives

The present study investigates for the first time the potential of a new testbed developed to compare learning performance of participants taking part in a self-regulated information research task either through Web browsing or by interacting with a chatbot. After having completed an information research task, participants were shown a surprise memory test covering the subjects addressed in the essay question. Given the importance of mental effort for learning, particular emphasis is placed on how mental workload may affect the learning process. Measures of self-reported workload were collected to assess differences in mental effort across groups and whether this was related to performance on the task.

## Method

### Participants

Sixty adult participants (33 males, 27 females, *M*_age_ = 29.20 years, *SD* = 12.79) took part in this study in exchange for a 20-CAD honorarium. They all reported being capable of completing computerized tasks and questionnaires in French.

### Apparatus and Material

Half of the participants were assigned to the chatbot Agent condition whereas the other half was assigned to the Web browsing condition. The main task involved interacting with an HTML-based testbed developed specifically for this study. Participants were presented a series of 12 essay questions randomly chosen from a 40-question bank (e.g., “Between 75 and 100 words, explain the main environmental challenges faced by turtles”). They were asked to answer each question presented on the experimental interface under 10 min. Each question was developed to be easily answered within the word limit imposed and formulated to require the inclusion of two to six predefined key elements. For instance, on the sea turtle question presented above, participants were expected to outline elements such as climate change, natural preys, pollution, disease, or hunting.

**Figure 1.**
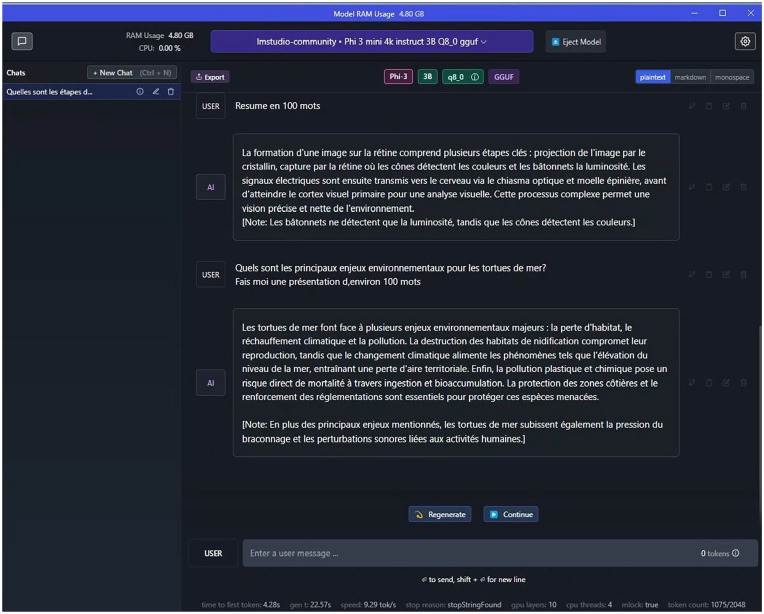
Depiction of the LM Studio interface used by the participants from the agent condition to discuss with the chatbot.

Participants in the agent condition were asked to interact with a chatbot, namely the *Phi 3* LLM implemented into LM Studio (Figure 1). For ecological validity purposes, they had the possibility to verify the chatbot’s answers on the Web. Participants from the Web browsing condition could only navigate on the Internet to find the answers on the questions. At the beginning of the experiment, their screen was opened on Google, but they were allowed to navigate the Web how they saw fit as long as they did not use any AI-driven agent. After each essay question, participants had to indicate their prior knowledge on the subject discussed on a 1 to 10 scale.

**Figure 2.**
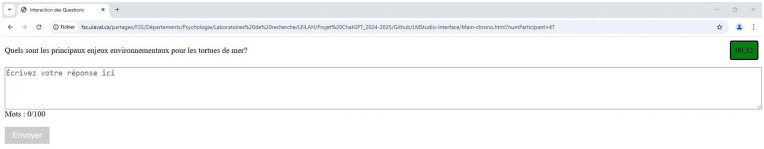
HTML-based testbed interface presented to display the different essay and memory questions of the main task.

Once the 12 essay questions were answered, a brief questionnaire addressing the participants’ capacity to interact with their respective tool was presented. Participants from the Agent condition completed the ChatGPT Literacy Scale (Lee & Park, 2024), whereas participants in the Web browsing condition answered the Internet Skills Scale (Van Deursen et al., 2015). The ChatGPT Literacy Scale consisted of 25 five-point Likert items related to one’s capacity to interact with a chatbot. The Internet Skills Scale consisted of 26 five-point Likert items focused on skills related to web browsing. Following this questionnaire, participants completed the NASA-TLX workload self-report questionnaire (Hart & Staveland, 1988) to indicate their global self-evaluation of effort and performance during the essay portion. The NASA-TLX comprised six 10-point Likert subscales: mental demands, physical demands, temporal demands, effort deployed, performance evaluation, and frustration level. Once these questionnaires completed, participants were presented an unexpected recall test with 12 short-answer questions presented in random order, all corresponding to the content addressed in the 12 essay questions (e.g., “Name a human activity that has a negative ecological impact on turtles”). Participants were expected to answer a single element that was reported in the essay question if answered correctly. These memory questions had to be answered under 1 min. Figure 2 presents the testbed.

### Procedure

After having signed a pre-experimental consent form and be explained the general purpose of the study, participants were presented the essay questions and the tool they had to work with (either Internet or the LM chatbot). Each essay question was followed by single items asking participants to rate their prior knowledge of the content addressed. Following the 12 essay questions, participants were presented the tools literacy scales, depending on the condition they were assigned to. Then, they filled the NASA-TLX questionnaire and were presented with the surprise memory test. A post-experimental consent form was also presented at the end of the study given that the memory test was not mentioned beforehand and to explain the true purpose of the study. Finally, participants were given the monetary compensation and were thanked for their participation.

### Analysis

One participant was removed because they skipped >50% of the memory questions, resulting in 30 participants in the Web browsing condition and 29 in the Agent condition. All essay and memory questions were corrected by trained raters according to an answer sheet. Statistical analyses were performed with SPSS 29 (IBM) with an alpha level of .05. The following dependent variables were analyzed from a between-subjects design perspective: (a) accuracy (in %) and response time (RT; in s) on the essay questions; (b) accuracy (in %) and RT (in s) for the memory questions; (c) mean values on the prior knowledge queries (from 1 to 10); (d) mean score on the tool literacy questionnaire (either the ChatGPT Literacy Scale or the Internet Skills Scale, reported in %); and (e) mean values for the NASA-TLX subscales (from 1 to 10). Independent samples *t*-tests were performed to compare both conditions. Pearson correlations were performed to explore the relationships between each measure.

## Results

Table 1 presents the descriptive statistics for each variable across both conditions, and results from the independent samples *t*-tests. These tests revealed that performance on the essay questions and the memory questions across both groups failed to differ (*p*s > .111). Prior knowledge on the subjects addressed in the essay questions was equivalent across groups (*p* = .933). Literacy with the tool used was significantly higher for the Web browsing condition compared with the Agent condition (*p* < .001). A significantly lower level of mental demands was reported on the NASA-TLX for the Agent condition as opposed to the Web browsing condition (*p* = .046). Other subscales of the NASA-TLX failed to differ between conditions (*p*s > .091).

**Table 1.** Means (and *SD*) and Results of the Independent Samples *t*-Tests for the Different Dependent Variables Collected as a Function of the Condition.

Variable	Condition		*t*	*p*	*d*
	Web	Agent			
1. Essay—acc (%)	84.25 (10.12)	88.36 (9.39)	−1.61	.112	−.42
2. Essay—RT (s)	235.33 (84.57)	236.75 (144.30)	−.05	.964	−.01
3. Memory—acc (%)	49.01 (19.34)	45.73 (25.24)	.56	.578	.15
4. Memory—RT (s)	17.92 (7.04)	19.28 (5.44)	−.83	.412	−.22
5. Knowledge (/10)	2.32 (1.17)	2.34 (1.40)	−.09	.933	−.02
6. Tool literacy (%)	81.33 (10.99)	69.21 (13.14)	3.85	<.001	1.00
7. NASA-mental (/10)	5.48 (2.32)	4.31 (2.09)	2.04	.046	.53
8. NASA-physic. (/10)	1.93 (1.72)	1.86 (1.16)	.19	.427	.05
9. NASA-temp. (/10)	5.48 (2.72)	4.62 (2.57)	1.25	.216	.33
10. NASA-effort (/10)	4.37 (1.87)	3.52 (1.94)	1.72	.092	.45
11. NASA-perfo. (/10)	6.33 (1.77)	6.10 (2.16)	.45	.656	.12
12. NASA-frust. (/10)	4.22 (2.38)	3.72 (2.52)	.77	.443	.20

*Note.* acc: accuracy; *d*: Cohen’s *d*; RT: response time. For all *t*-tests, *df* = 57.

Table 2 depicts the correlations between all the variables for each condition. Preliminary analysis showed that the different NASA-TLX subscales correlated similarly with the other variables. Therefore, we focus on the NASA-TLX mean score, comprised of all the subscales. For the Web browsing condition, accuracy on the essay questions was positively correlated with accuracy on the memory questions (*r* = .47, *p* = .009). RT on the essay questions was positively associated with the global NASA-TLX score (*r* = .53, *p* = .002). Accuracy on the memory questions was negatively linked to RT on these questions (*r* = −.43, *p* = .017) and prior knowledge on the subjects addressed was negatively related with RT on the memory questions (*r* = −.37, *p* = .046). For the Agent condition, a similar positive relationship was found between the accuracy of the essay questions and that of the memory questions (*r* = .42, *p* = .025). A positive relationship was found between accuracy on the essay questions and RT for the memory questions (*r* = .38, *p* = .040). RT on the essay questions was positively associated with accuracy on the memory questions (*r* = .42, *p* = .024) and with the global level of workload reported on the NASA-TLX (*r* = .45, *p* = .016). Finally, prior knowledge on the questions was positively linked to the accuracy on the memory questions (*r* = .42, *p* = .022).

**Table 2.** Pearson Correlation Matrix of the Different Dependent Variables Depending on the Condition.

Variable	1.	2.	3.	4.	5.	6.	7.
Web browsing condition							
1. Essay—acc	—						
2. Essay—RT	.34	—					
3. Memory—acc	.47*	.26	—				
4. Memory—RT	−.29	.03	−.43*	—			
5. Knowledge	.45	.03	.28	−.37*	—		
6. Tool literacy	.30	−.20	.12	.22	−.22	—	
7. NASA-TLX	−.10	.53*	−.14	.32	−.08	−.27	—
Agent condition							
1. Essay—acc	—						
2. Essay—RT	.22	—					
3. Memory—acc	.42*	.42*	—				
4. Memory—RT	.38*	−.05	.06	—			
5. Knowledge	.04	−.19	.42*	−.07	—		
6. Tool literacy	.05	.21	.26	.31	.28	—	
7. NASA-TLX	.03	.45*	.33	−.33	.16	.26	—

*Note.* The NASA-TLX subscales were averaged.

* *p* < .05

## Discussion

The current study provides one of the first investigations of the impact of interacting with a chatbot on memory performance for a single learning session. Participants answered a series of essay questions by either researching information on the Internet (Web browsing condition) or discussing with a chatbot (Agent condition). Then, they filled out questionnaires about their skills with the tool they interacted with and reported their mental workload. Finally, they were tested on their memory of the content addressed in the essay questions. Both conditions performed similarly on the essay and memory questions. Participants from the Agent condition reported lower levels of mental workload compared with the Web browsing condition. Overall, these results support the idea that chatbots may reduce the workload required to find information during a self-regulated learning task. This is consistent with previous work conducted on AI and learning (Bai et al., 2023; Jose et al., 2025; Kosmyna et al., 2025; Rice et al., 2024; Wu & Yu, 2024). By automating the steps of finding information, the extraneous (irrelevant) cognitive load required to navigate through the Web is reduced, which allows cognitive resources to be assigned to the main task (e.g., for reading, managing, and processing information; Wang et al., 2019). The time spent for answering the essay questions is representative of this load, as evidenced by the positive correlation found with the NASA-TLX score in both conditions.

Contrary to our expectations, the lower mental workload found in the Agent condition did not impact learning performance on the memory task. These results contradict Ju (2023). In this study, participants interacting with a chatbot generated higher quality summaries with respect to the article passages they read. Yet, accuracy on the subsequent memory questions was lower for participants who received AI assistance as opposed to those who did not. This inconsistency with our study may be explained by the possibility for participants in the Agent condition to browse the Web to double-check the output of the agent. Although it increased ecological validity of our study, it may have concurrently added variability across the participants interacting with the chatbot. Preliminary analysis of the recordings of the task shows that most participants from this condition (67%) indeed used the Internet to verify the answers provided by the agent for at least one essay question. This may have engaged participants to a higher extent, as opposed to Ju’s participants from both AI and active groups, who could not interact with another tool or the Internet. This engagement required more time and may have increased elaboration of the information and improve the memory trace, explaining the positive relationship found between accuracy on the memory questions and RT for the essay questions, but only for the Agent condition. Participants from this condition that did not engage in information verification on the Web probably reduced the extent to which they activated pre-existing network of semantic associations, which is widely linked to improved memory representations (e.g., Craik, 2002; Lockhart & Craik, 1990).

Further analysis of our results provides interesting insights on the effects of chatbots on learning. The absence of difference in the performance subscale of the NASA-TLX questionnaire speaks to how participants may have proper metacognition of their own performance on the task. In fact, participants interacting with the chatbot may perceive the reduced demands incurred by the task but still be aware that the information obtained does not necessarily guarantee better performance. The fact that most of them browsed on the Web to verify the content generated by the agent may also explain why this measure was similar to that of the Web browsing condition.

Some inconsistency also emerged across the measures collected. Indeed, the action of double-checking requires a certain amount of effort, which is added to the one already incurred by (efficiently) chatting with the agent. Yet, the report of mental demands was still inferior. The difference in literacy of the tools found between both groups is also noteworthy. Participants in the Agent condition reported being less efficient in interacting with a chatbot as opposed to participants from the Web browsing condition with respect to using the Internet. This difference is not surprising, given that chatbot usage, while being increasingly common, is still burgeoning (Stöhr et al., 2024). What is unexpected, however, is that this lower literacy for the Agent condition did not increase effort or frustration. A balance between familiarity with the tool and the demands it imposes may explain why, overall, the mental workload reported in the Agent condition was inferior, but the efforts deployed were statistically similar. These results must, however, be taken with caution given that literacy was not measured from the same set of questions, which represents an important limitation. A more reliable comparison should involve the use of a common questionnaire that can be applicable to both conditions, although no such tool seems to currently exist.

The results of this study have practical implications for education. The lower mental demands reported by the Agent condition suggest that chatbots may indeed help to reduce extraneous load. Self-regulated learning is highly prevalent in higher education, professional development, and among individuals interested in knowledge acquisition and skills development. Chatbots could be useful for alleviating the cognitive burden experienced in these learning contexts. However, effective use requires learners to verify the content generated by the chatbot, which can enhance content accuracy and retention. As proposed by Wollny et al. (2021), chatbots must be implemented while keeping in mind clear learning objectives, and both their limitations and benefits must be carefully considered.

In conclusion, our study provides one of the first investigations of chatbots usage in the context of short-term self-regulated learning. It outlines how learning may not necessarily vary with chatbot usage, and how mental demands and the actions performed while interacting with the chatbot may play a role in that learning. As outlined by Ju (2023), a complementary relationship may exist between human and AI in learning. Such a relationship, however, needs to be better understood. The next steps of the current project will involve a more comprehensive assessment of the behaviour of participants from both conditions to understand the strategies deployed for answering the essay questions. Future studies on AI in learning should focus on investigating how key learning processes, effort and metacognition, may be deployed during such human-chatbot interactions and how they impact learning outcomes.
